# FAM83A antisense RNA 1 (*FAM83A-AS1*) silencing impairs cell proliferation and induces autophagy via MET-AMPKɑ signaling in lung adenocarcinoma

**DOI:** 10.1080/21655979.2022.2081457

**Published:** 2022-05-29

**Authors:** Huijie Zhao, Yinghan Wang, Xing Wu, Xaofei Zeng, Baoyue Lin, Shengmin Hu, Shenglin Zhang, Yu Li, Zhiqing Zhou, Yujie Zhou, Changzheng Du, David G. Beer, Shengbin Bai, Guoan Chen

**Affiliations:** a School of Medicine, Southern University of Science and Technology, Shenzhen, Guangdong, China; b School of Life Science, Southern University of Science and Technology, Shenzhen, Guangdong, China; c Department of Surgery, University of Michigan Medical School, Ann Arbor, MI, USA; d Department of Histology and Embryology, Basic Medical College, Xinjiang Medical University, Urumqi, China

**Keywords:** Lung adenocarcinoma, lncRNA, FAM83A-AS1, MET, autophagy

## Abstract

Studies demonstrate that long non-coding RNAs (lncRNAs) play vital roles in cancer progression. However, the expression pattern and molecular mechanisms of lncRNA *FAM83A-AS1* in lung cancer remain largely unclear. Here, we analyzed *FAM83A-AS1* expression in lung cancer tissues from three RNA-sequencing (RNA-Seq) datasets and validated these results using quantitative real-time reverse transcription polymerase chain reaction (qRT-PCR) in an independent set of lung adenocarcinoma. Cell proliferation, migration, invasion, and autophagy were analyzed after knockdown *FAM83A-AS1* with siRNAs. The underlying molecular mechanisms of *FAM83A-AS1* were performed by Western blot, qRT-PCR, and RNA-seq analysis. We found that *FAM83A-AS1* was up-regulated in lung cancer and elevated expression was associated with poor patient survival. These results were confirmed using RT-PCR in an independent set of lung cancer. Functional study indicated that *FAM83A-AS1* knockdown reduced cell proliferation, migration, invasion, and colony formation in cancer cells. *FAM83A-AS1* silencing induced autophagy and cell cycle arrest at G2. Mechanistically, serval oncogenic proteins such as EGFR, MET, PI3K, and K-RAS were decreased upon *FAM83A-AS1* silencing, while phosphor AMPKα and ULK1 were increased. Based on the above results, we believe that *FAM83A-AS1* may have potential as a diagnosis/prognosis marker and its oncogenic role and autophagy regulation may be through MET-AMPKα signaling, which could lead to potential targeting for lung cancer therapy.

## GRAPHICAL ABSTRACT

**Figure:**
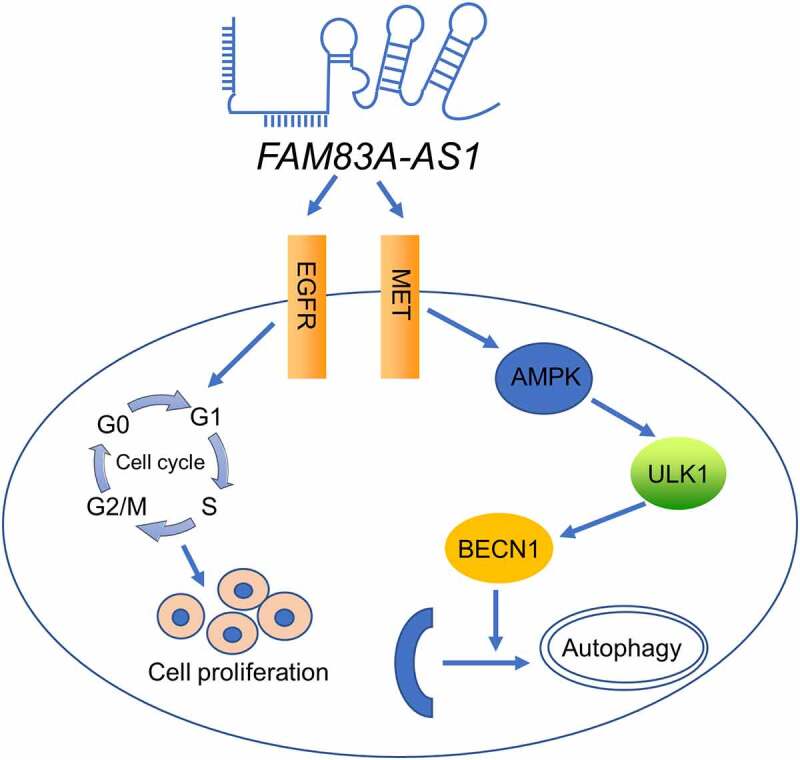

## Highlights

- *FAM83A-AS1* was up-regulated in lung cancer and elevated expression was associated with poor patient survival.
- *FAM83A-AS1* knockdown reduced cell proliferation, migration, invasion, colony formation and induced autophagy and cell cycle arrest at G2.
- Mechanistically, serval oncogenic proteins such as EGFR, MET, PI3K, and K-RAS were decreased upon *FAM83A-AS1* silencing, while phosphor AMPKα and ULK1 were increased.
- *FAM83A-AS1* may have potential as a diagnosis/prognosis marker and its oncogenic role and autophagy regulation may be through MET-AMPKα signaling.

## 1. Background

Lung cancer ranks second in morbidity and first in mortality worldwide [1]. The two major types are non-small cell lung cancer (NSCLC) and small cell lung cancer (SCLC). NSCLC includes lung adenocarcinoma (LUAD), lung squamous cell carcinoma (LUSC), and lung large cell carcinoma (LLC). Lung cancer makes up approximately a quarter of all cancer-related deaths [1]. When most patients are diagnosed, 80% are at an advanced stage where the prognosis is relatively poor [2]. In recent years, breakthroughs in molecular diagnosis and targeted therapy of lung cancer have been made [3–5], but the five-year overall survival remains at only 20% [1].

LncRNAs are one type of RNA with nucleotides longer than 200 base pairs and are usually not translated into protein [6]. Studies have suggested that lncRNAs play critical roles in tumor development and progression via different mechanisms [7–10]. For example, lncRNA *PVT1* stimulates tumor growth via miR-143/HK2 signaling in gallbladder tumor [7]. LncRNA *DNM3OS* contributes to tumor metastasis through regulation of the epithelial-to-mesenchymal transition in ovarian cancer [9]. Knockdown of lncRNA *MIR22HG* induces cell survival through YBX1/MET signaling in LUAD [11]. With the advantage of applying next-generation sequencing, we have uncovered several dysregulated lncRNAs in lung cancer [11–17]. However, the mechanisms of their oncogenic actions are not completely understood.

There are several reports regarding lncRNA *FAM83A-AS1* in cancer [18–25]. It was reported *FAM83A-AS1* expression is higher in esophageal cancer and can promote cancer progression via binding of miR-495-3p [18]; whereas knockdown of *FAM83A-AS1* impaired cell growth and induced cell apoptosis through binding of NOP58 in liver cancer [19]. *FAM83A-AS1* was suggested to affect lung cancer cell invasion and migration through miR-150-5p/MMP14 signaling [20] or miR-495-3p [23] and may promote lung cancer progression through increasing FAM83A expression [21,24] or glycolysis via regulating HIF-1α degradation [26]. However, the detailed mechanism of *FAM83A-AS1* in lung cancer remains uncertain.

This work aims to explore the function and mechanism of *FAM83A-AS1* in non-small cell lung cancer. By combining our data with publicly available RNA-Seq data, we found that *FAM83A-AS1* was up-regulated in lung cancer, and elevated expression of *FAM83A-AS1* was significantly related to poor patient survival. Further assays results revealed that cell proliferation was decreased, and autophagy was induced upon *FAM83A-AS1* silencing. We hypothesized that *FAM83A-AS1* functions in lung cancer cells may be through MET-AMPKα signaling and tested the hypothesis by more in-depth experiments. The goal of this research was to explore the potential target or biomarker for lung cancer therapy.

## 2. Methods

### 2.1. Cell lines

We selected H1299, H838, H1975, H1650 and, PC9 cell lines to carry out all experiments. The cell culture medium contained RPMI 1640 (Invitrogen), 10% fetal bovine serum, and 1% penicillin-streptomycin. Cell culture was carried out using standard cell culture conditions at 37C°, 5% CO2, and appropriate humidity. All cell lines were purchased from the American Type Culture Collection and screened for mycoplasma contamination. Genotyping was performed at the Guangzhou Cellcook Biotech Co., Ltd. (Guangzhou, China), and the results are attached in supplementary files.

### 2.2. Tissue samples

Clinical samples included lung cancer tissues and paired non-tumoral lung tissues from patients undergoing curative cancer surgery during the period from 1992 to 2015 at the University of Michigan Health System. The study was reviewed and approved by the University of Michigan Institutional Review Board and Ethics Committee. All of the patients were without preoperative chemoradiotherapy treatment and had provided informed consent. The median follow-up time was 8.5 years. Surgical samples were quickly frozen in liquid nitrogen and maintained at −80°C before both cryostat sectioning and pathological analysis and RNA extraction. All patient samples used in this study by qRT-PCR validation (101 LUADs) were reported in our previous studies [11,12].

### 2.3. RNA extraction and qRT-PCR

Total RNAs were extracted using the RNeasy Mini kit (Qiagen) according to the manufacturers’ instructions. The qRT-PCR reactions were carried out with the Power SYBR Green master mix, which was purchased from Life Technology Inc. The cDNA was amplified by real-time quantitative PCR using an ABI System (Applied Biosystems) following the manufacturer’s instruction. Each reaction used contained 10 ng cDNA in a final reaction volume of 15 µL. The PCR primers of *FAM83A-AS1* were: forward, 5’-GGAAGCAGGGCTCTTCAGTT, and reverse, 5’-AGGGCCGTCTGTGTTTACTG. GAPDH served as housekeeping normalization control to ensure equal loading.

### 2.4. Published RNA-Seq data collections

RNA-seq data sets were downloaded from previously published papers [12,27–30], which included UM (University of Michigan) [29], Seo [28], TCGA [27], and CCLE cell lines [30] data portals. Lung samples consist of six normal lung tissues, 67 LUAD, 36 SCC, and 10 LLC in the UM dataset [29]; 77 normal lung tissues and 85 LUADs in the Seo dataset [28]; 73 normal lung tissues and 309 LUADs in the TCGA dataset [27]. Fragment per kilobase million mapped reads (FPKM) was calculated to identify transcripts and measure their relative expression levels [29,31].

### 2.5. siRNA-mediated knockdown

PC-9, H838, and H1299 cells were plated at the appropriate cell density and were separated into the non-target control group and *siFAM83A-AS1* group. *FAM83A-AS1* siRNA sequences were all located in the first exon of this lncRNA. A mixture of 10 nM experimental siRNA oligonucleotides or non-targeting controls, Lipofectamine® RNAiMax Reagent (Invitrogen, USA) and Opti-MEM were included in each respective 96-well at the appropriate proportion. Knockdown of siRNAs was performed 24 h after plating and qRT-PCR was used to detect the knockdown efficiency.

### 2.6. Cell proliferation assay

H838 and H1299 were plated at the desired concentration. Once cells were attached to the substratum, the medium was changed to antibiotic-free culture medium, transfected with 10 nM negative control siRNA oligonucleotides or *siFAM83A-AS1* oligonucleotides. Transfection was implemented as described above. The proliferation rate was measured 72 h after transfection. Cell viability was measured using WST-1 reagent (Roche). The percentage of cellular viability was calculated as a percentage relative to the control group.

### 2.7. Colony formation assay

Forty-eight hours after treatment with *FAM83A-AS1* siRNAs, cancer cells were suspended with trypsin-treatment and then plated in 6-well plates (500 cells per well). After 10 to 14 days of incubation, 20% methanol was used to fix the clones for 20 minutes and 0.1% crystal violet was used to stain the clones for 30 minutes. After washing away the excess crystal violet, the colonies were photographed and counted. More than 50 cells were regarded as a colony.

### 2.8. Migration and invasion

For invasion assays, Matrigel (BD company) was maintained at 4°C overnight for dissolution before dilution with cold serum-free RPMI 1640 (Matrigel: RPMI 1640 = 1:8). Basement membrane matrix Boyden chambers (8-mm pore size) were purchased from BD Company and 100 ul Matrigel was added to each upper chamber. Resuspended cells in serum-free RPMI-1640 were adjusted to a cell density of 1–5 × 105/ml, with total cells being 2–10 × 10^4^ per well (H1299: 5 × 10^4^ per well; H838: 10 × 10^4^ per well). 200 μl of RPMI-1640 with cells was added into the upper chamber and 600 μl of 20% FBS-containing RPMI-1640 was added into the lower chamber. Cells were incubated for 48 h at 37°C, then fixed, stained, and counted. Five fields are randomly selected for counting the migrated cell numbers. For invasion assays, the only difference was that no Matrigel was included in the basement membrane matrix Boyden chambers.

### 2.9. Flow cytometry for cell cycle analysis

Forty-eight hours after treatment with *FAM83A-AS1* siRNAs, cancer cells were suspended with trypsin treatment, washed twice with ice-cold phosphate buffer saline (PBS) and the supernatant removed after centrifugation at 1000 × r for 10 min. The cells were fixed with 70% ice-cold ethanol and then stored overnight at 4°C. After washing with PBS, 1 ml of propidium iodide (PI) staining solution (containing 0.1% (v/v) Triton X-100, 10 μg/mL PI, and 100 μg/mL DNase-free RNase A in PBS) was added to the tubes and incubated for 30 min at room temperature in the dark. Flow cytometer was used to measure the cell cycle. The experimental operation was as gentle as possible to reduce cell damage.

### 2.10. Autophagy assay

The Premo Autophagy Tandem Sensor RFP-GFP-LC3 (Life Technologies, P36239) was used for detecting autophagic flux. Cells were plated at the required concentration, transfected at 24 h, and adenovirus added at 48 h. The culture medium containing adenovirus was replaced with new medium after 6–8 h. Autophagy fluorescence was observed using an Olympus IX71 microscope 24 and 48 h after incubation. Autolysosomes (red dots in fusion images) and autophagosomes (yellow dots in fusion images) were counted in at least 100 cells in a 200X field.

### 2.11. Western blot

Cells were lysed with RIPA lysis buffer on ice, and the supernatant was collected following centrifugation for 20 min at 14000 g. The extracted protein was quantified, and 10 mg protein per sample was separated according to their molecular weight by 10% polyacrylamide gel electrophoresis. Proteins were transferred onto polyvinylidene difluoride membrane and blocked for 1 h with 5% bull serum albumin (BSA) before adding primary antibodies and incubating overnight at 4°C. Antibodies and concentration were as follows: phospho-AMPKα (Cell Signaling #2535s, at a 1:1000 dilution), phospho-ULK1 (Ser555) (Cell Signaling #5869, at a 1:1000 dilution), total AMPK (Cell Signaling #5832s, at a 1:1000 dilution), phospho-BECN1 (Cell Signaling #35955, at a 1:1000 dilution), LC3B (Cell Signaling #3868s, at a 1:1000 dilution), GAPDH (Cell Signaling #5174s, at a 1:5000 dilution), total MET (Cell Signaling #8198, at a 1:1000 dilution), total EGFR (Cell Signaling #4267s, at a 1:1000 dilution), phospho-p44/42 Erk1/2 (Cell Signaling #9101s, at a 1:1000 dilution), p44/42 Erk1/2 (Cell Signaling #9102s, at a 1:1000 dilution), phospho-MET (Cell Signaling #3077, at a 1:1000 dilution), p62 (Sequestosome 1, SQSTM1, Cell Signaling #5114, at a 1:1000 dilution), phospho-s6k (p70 S6 Kinase, Cell Signaling #2708, at a 1:1000 dilution), Phospho-p38 (Cell Signaling #9211s, at a 1:1000 dilution), PI3K (Cell Signaling #4257s, at a 1:1000 dilution), K-RAS (Cell Signaling #53270s, at a 1:1000 dilution). The second antibody (1:5000 dilution) was then added the following day and incubated at room temperature for 1 h. The membranes were developed using ECL (Millipore Sigma) and exposed with the ChemiDoc MP Imaging System (BIO-RAD). The detected instrument was ChampChemi 610 plus, a fully automated chemiluminescence instrument (Sage creation, Beijing, China).

### 2.12. RNA-seq analysis

Cell palates were collected after being treated with *FAM83A-AS1* siRNAs and control siRNAs at 48 hours in lung cancer cell lines. Total RNA was extracted using a Trizol reagent kit (Invitrogen, Carlsbad, CA, USA) according to the manufacturer’s protocol. The cDNA library was sequenced using Illumina Novaseq6000 by Gene Denovo Biotechnology Co. (Guangzhou, China).

### 2.13. Statistical analysis

Data were collected and statistically analyzed using GraphPad Prism 6 and R software. Receiver Operating Characteristic (ROC) curves of the results were created and the area under the curve (AUC) was calculated to describe diagnostic efficacy. The Kaplan–Meier method was utilized to plot survival curves of patients and a log-rank test was used to compare survival times between different groups. The expression levels of *FAM83A-AS1*, cell proliferation, migration, invasion, and colony formation were evaluated with unpaired Student’s t-test. The cutoff for considering a significant difference was established with a *P*-value < 0.05. KEGG [32] and Gene Ontology (GO) [33] related databases were used for pathway and biology process analysis.

## 3. Results

In this study, we have found that *FAM83A-AS1* expression was higher in lung cancer tissues as compared to normal lung tissues, and higher expression of *FAM83A-AS1* was significantly correlated to unfavorable patients survival. *In vitro* study indicated that *FAM83A-AS1* silencing impaired cell proliferation, migration, invasion, and colony formation in lung cancer cells. *FAM83A-AS1* knockdown induced cell autophagy and cell cycle arrested. Mechanistically, serval oncogenic proteins including EGFR, MET, PI3K, and K-RAS were decreased upon *FAM83A-AS1* silencing, while phosphor AMPKα and ULK1 were increased. We postulated that *FAM83A-AS1* played an oncogenic role in lung cancer progression and cell autophagy regulation may be through MET-AMPKα signaling.

### 3.1. FAM83A-AS1 is overexpressed in lung cancer and high expression is associated with poor patient survival

**Figure 1.**
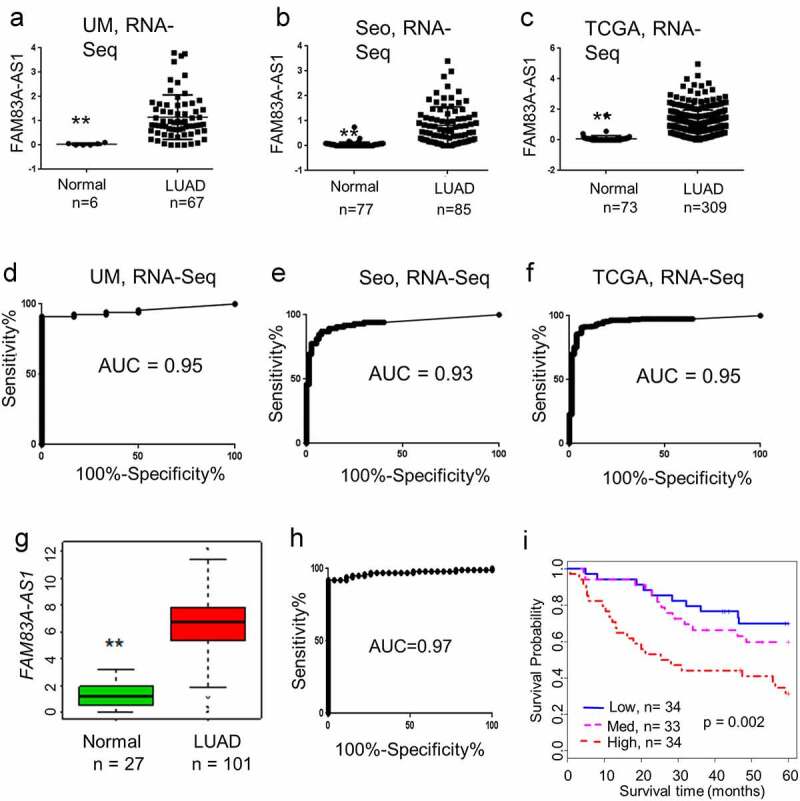
*FAM83A-AS1* is overexpressed in LUAD and unfavorable for patient survival. (a–c), *FAM83A-AS1* expression levels are shown as Scatter plots. RNA-Seq (6 normal samples and 67 LUADs) results of UM, Seo, and TCGA show that *FAM83A-AS1* is up-regulated in lung adenocarcinoma (log2 of FPKM value as y-axis, groups and number of samples in x-axis; ** represents *p < *0.01, LUAD vs. normal); (d–f), ROC curves and corresponding AUC values of *FAM83A-AS1* in UM (6 N vs 67 LUAD), Seo (77 N vs 85 LUAD), and TCGA (73 N vs 309 LUAD) RNA-Seq data sets; (g), Box plot indicating *FAM83A-AS1* expression is higher in tumor in an independent validation set (101 LUAD and 27 normal lung tissues) verified by RT-PCR (fold-change of FPKM to mean of all tissues as y-axis, loading control of PCR is ACTB; ** represents *p* < 0.01 by t test, LUAD vs. normal); (h), ROC analysis revealed that *FAM83A-AS1* was a good predictor (AUC is 0.97) for classifying the normal lung tissues and LUAD; (i), Increased *FAM83A-AS1* expression is correlated with poor patient survival in the validation set indicating (log-rank test, *p* = 0.002).

RNA-Seq data was acquired from previously published papers including the Seo, TCGA, and UM data sets [12,27–29]. FPKM values for lncRNA expression were calculated to identify transcripts and measure their relative expression levels. The results showed that *FAM83A-AS1* was up-regulated in LUAD (Figure 1(a–c)). ROC curves of the results were created based on the expression of *FAM83A-AS1* in LUAD vs. normal lung and the AUC in these three data sets were 0.95, 0.93, and 0.95, respectively (Figure 1(d–f)). To verify these results, *FAM83A-AS1* expression from an independent cohort of lung adenocarcinoma tissues including 101 LUADs and 27 normal lung samples was analyzed by qRT-PCR. The *FAM83A-AS1* expression level was higher in LUAD relative to normal lung tissues and the AUC was 0.97 (Figure 1(g,h)). Further, the high expression of *FAM83A-AS1* was significantly related to poor prognosis in patients with lung cancer (Figure 1(i)). Collectively, these data indicate that *FAM83A-AS1* is overexpressed in LUAD and unfavorable for patient survival. *FAM83A-AS1* may have potential as a diagnosis/prognosis marker for lung cancer.

### 3.2. FAM83A-AS1 expression is higher in different types of lung cancers and is localized in the cytoplasm

**Figure 2.**
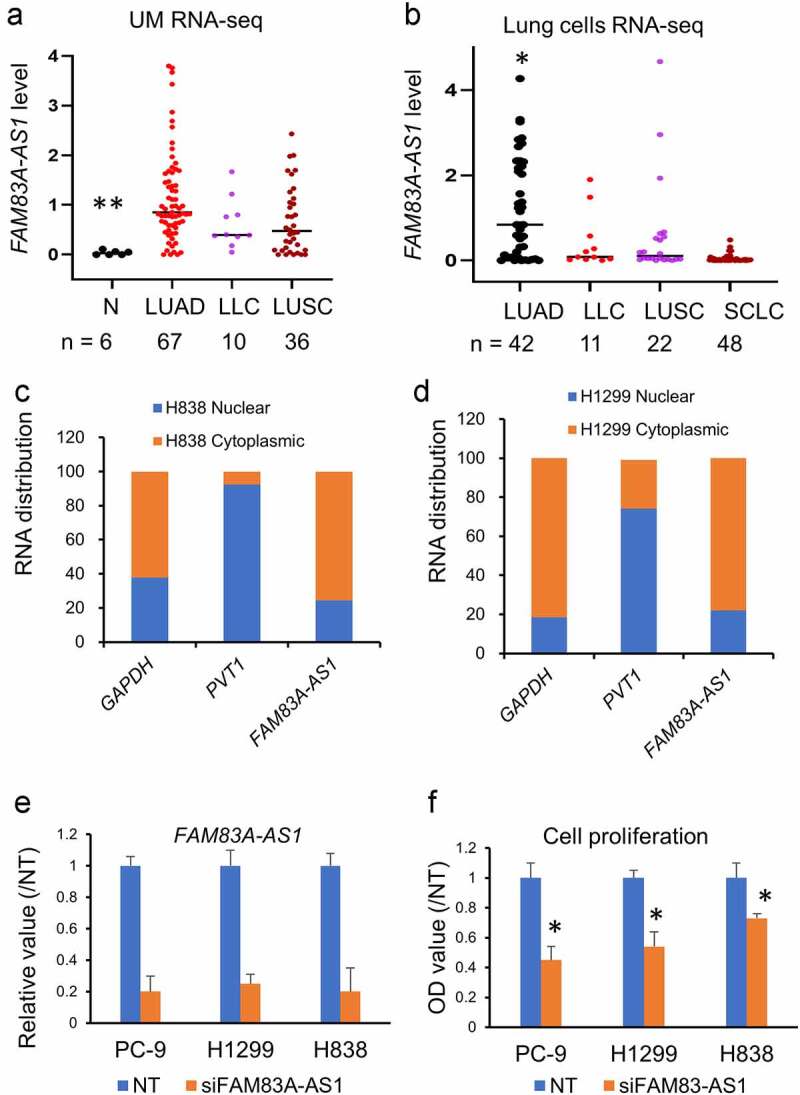
*FAM83A-AS1* expression in different type of lung cancers, cellular location and cell proliferation. (a), *FAM83A-AS1* expression is higher in lung adenocarcinoma (LUAD), large cell lung cancer (LLC) and squamous cell lung cancer (LUSC) as compared to normal lung tissues (**, *p* < 0.01 by t test); (b), *FAM83A-AS1* expression in different type of lung cancer cell lines (CCLE RNA-Seq data set). *FAM83A-AS1* is higher in AD as compared to LLC, LUSC and small cell lung cancer (SCLC) (*, *p* < 0.05 by t test); (c, d), The location of *FAM83A-AS1* was shown by qRT-PCR in H1299 and H838 cells. PVT1 snRNA as nuclear control and GAPDH was used as cytoplasmic control. *FAM83A-AS1* is mainly in cytoplasm (61% – 77%). (e), Knockdown efficiency of *FAM83A-AS1* siRNA at 48 h in PC-9, H1299 and H838 cell lines verified by qRT-PCR. GAPDH expression selected as the loading control; (f), Cell proliferation is decreased after *FAM83A-AS1* siRNAs treatment in lung cancer cells. * *p* < 0.05 by t test.

RNA-Seq results of lung cancer samples from UM cohort, which included 6 normal lung tissues, 67 lung adenocarcinoma tissues, 10 large cell lung cancer tissues, and 36 lung squamous cell carcinoma tissues [12], showed that *FAM83A-AS1* expression is not only higher in lung adenocarcinoma but also higher in LLC and LUSC as compared to normal lung tissue. Of these, the highest expression was found in LUAD (Figure 2(a)). We then analyzed the expression of *FAM83A-AS1* in different lung cancer cell lines [30], which included 42 lung adenocarcinoma cell lines, 11 large cell lung cancer cell lines, 22 lung squamous cell lines, and 48 small cell lung cancer cell lines. *FAM83A-AS1* remained highly expressed in LUAD, LUSC, and LLC, but was lower in SCLC (Figure 2(b)). To determine the cellular location of *FAM83A-AS1*, and thus its potential site of action, qRT-PCR was performed in H1299 and H838 cells. GAPDH is used as cytoplasmic control and PVT1 lncRNA as nuclear control. The results showed that *FAM83A-AS1* was primarily expressed in the cell cytoplasm (61–77%) (Figure 2(c,d)), suggesting its action may occur in the cytoplasm.

### 3.3. FAM83A-AS1 knockdown impairs cell proliferation, colony formation, cell migration and invasion

**Figure 3.**
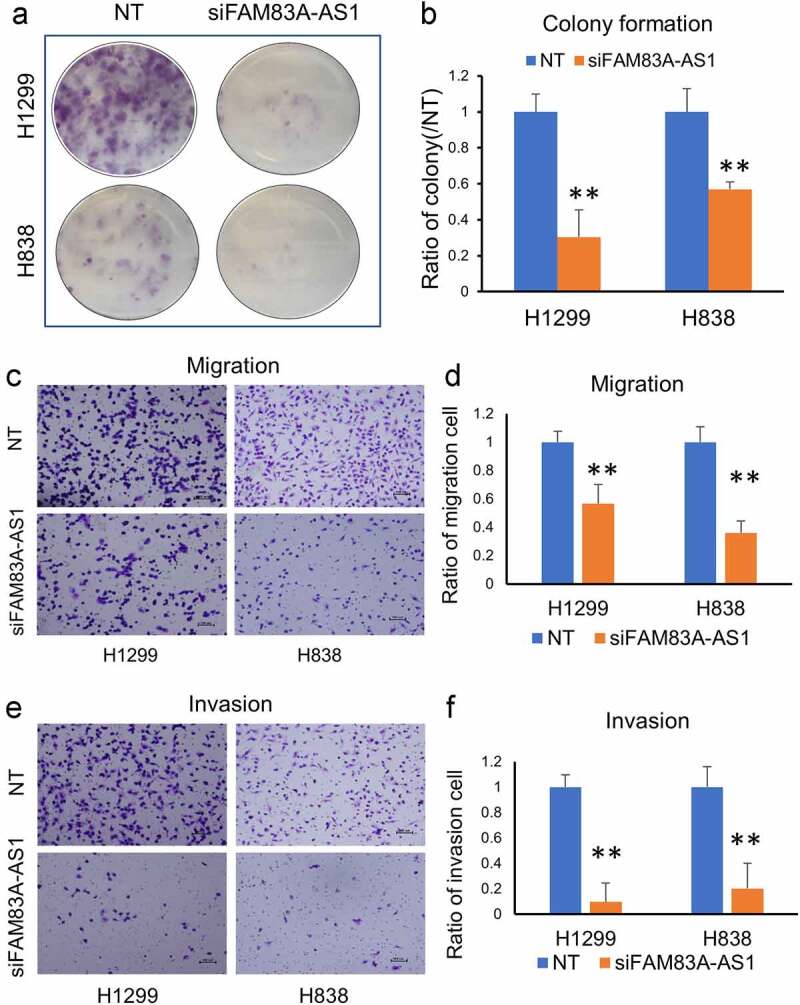
*FAM83A-AS1* knockdown impairs colony formation, cell migration and invasion. (a, b), Knockdown of *FAM83A-AS1 by* siRNA impaired cell colony formation in H838 and H1299 cells, B is the relative quantified value from A image; (c, d), Silencing of *FAM83A-AS1 by* siRNA reduced cell migration in H838 and H1299 cells (10X), D is the relative quantified value from C, 5 fields are randomly selected for counting the migrated cell numbers. (e, f), *FAM83A-AS1* knockdown by siRNA impaired cell invasion in H838 and H1299 cells (10X), F is the relative quantified value from E, 5 fields are randomly selected for counting the cell numbers.

*FAM83A-AS1* knockdown efficiency by siRNAs in PC-9, H838, and H1299 cell lines was examined by qRT-PCR. We found that *FAM83A-AS1* was decreased by 80% at 48 h after siRNAs treatment (Figure 2(e)). After *FAM83A-AS1* siRNA treatment, cell proliferation was decreased in PC-9, H838, and H1299 cells (*p*< 0.05) (Figure 2(f)). The colony formation ability and cell cycle were measured at 48 h after siRNAs transfection. The results showed that colony formation ability was decreased in H1299 and H838 cell lines (*p*< 0.01) (Figure 3(a,b)). To investigate the effect of *FAM83A-AS1* on cancer cell metastatic potential, cell invasion and migration assays were completed in H1299 and H838 cell lines. After silencing of *FAM83A-AS1* by siRNAs, cellular migration and invasion ability were significantly decreased in these two cell lines (Figure 3(c–f)), suggesting that *FAM83A-AS1* could promote lung cancer invasiveness or metastasis.

### 3.4. FAM83A-AS1 silencing causes cell cycle arrested and induces autophagy

**Figure 4.**
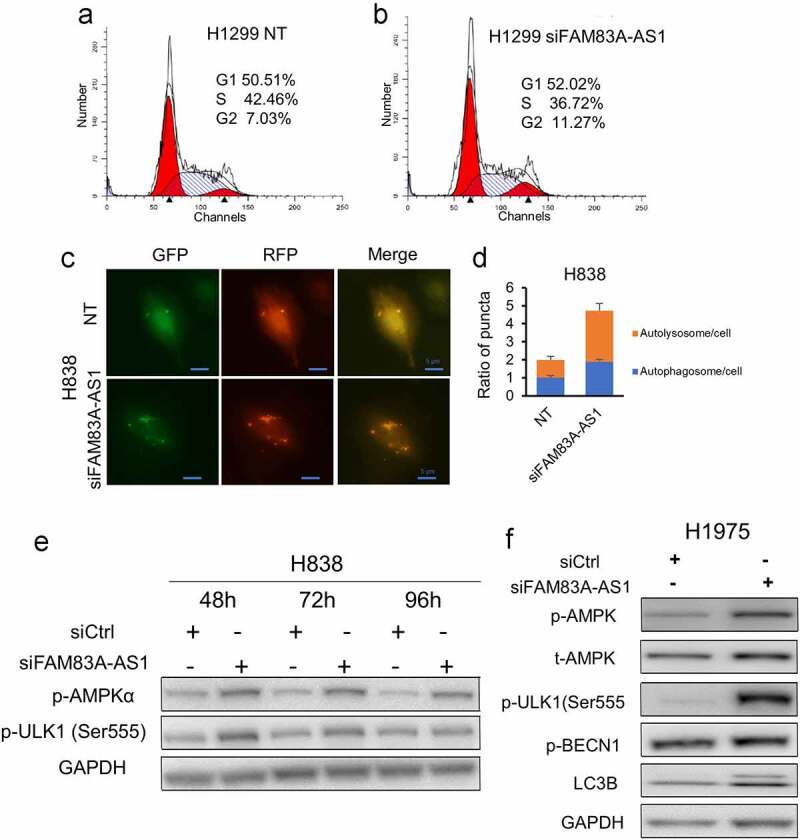
*FAM83A-AS1* knockdown induces cell cycle arrest and autophagy. (a, b), Silencing of *FAM83A-AS1* caused cell cycle arrested at G2 phase in H1299 cells. (c, d), H838 cells were treated with *FAM83A-AS1* siRNAs for 48 h and infected with Premo Autophagy Tandem Sensor RFP-GFP-LC3B for 24 h. Cells were visualized alive with fluorescence microscope. Autophagosomes and autolysosomes in each 200X field were counted, at least 100 cells were counted for siRNA or NT treatment cells. Autophagic flow was increased upon *FAM83A-AS1* silencing. Scale bar: 5 µm. D is the relative quantified value from image C. (e, f), The autophagy related proteins and phosphor AMPK, ULK, BECN1 and LC3B II were increased after silencing of *FAM83A-AS1* by siRNAs in H838 and H1975 cells.

Since *FAM83A-AS1*knockdown decreases cell proliferation, we want to know which phase was affected. Flow cytometer was performed upon *FAM83A-AS1*knockdown. We found that the cell cycle was arrested at the G2 phase in H1299 cells after the knockdown of *FAM83A-AS1* (Figure 4(a,b)), suggesting *FAM83A-AS1* involves cell cycle regulation in lung cancer.

Autophagy is a significant cellular degradation process and is closely related to cancer progression. In this study, autophagic flux was measured using Premo Autophagy Tandem Sensor RFP-GFP-LC3. In Figure 4(c), the green signal represents GFP fluorescence, red puncta represent autolysosomes, and yellow puncta represent autophagosomes. Following the silencing of *FAM83A-AS1* by siRNA, both autophagosomes and autolysosomes were increased in H838 cells (Figure 4(c,d)), indicating that *FAM83A-AS1* inhibits autophagy as one of the mechanisms in regulating cell death signaling in lung cancer.

Next, we examined the major autophagy-related proteins and found that phosphor-AMPKα, phosphor-ULK1, phosphor-BECN1, and LC3B II were increased (Figure 4(e,f)), suggesting *FAM83A-AS1* regulating autophagy may through AMPKα phosphorylation.

### 3.5. RNA-seq analysis showing FAM83A-AS1 is involved in multiple cancer pathways

**Figure 5.**
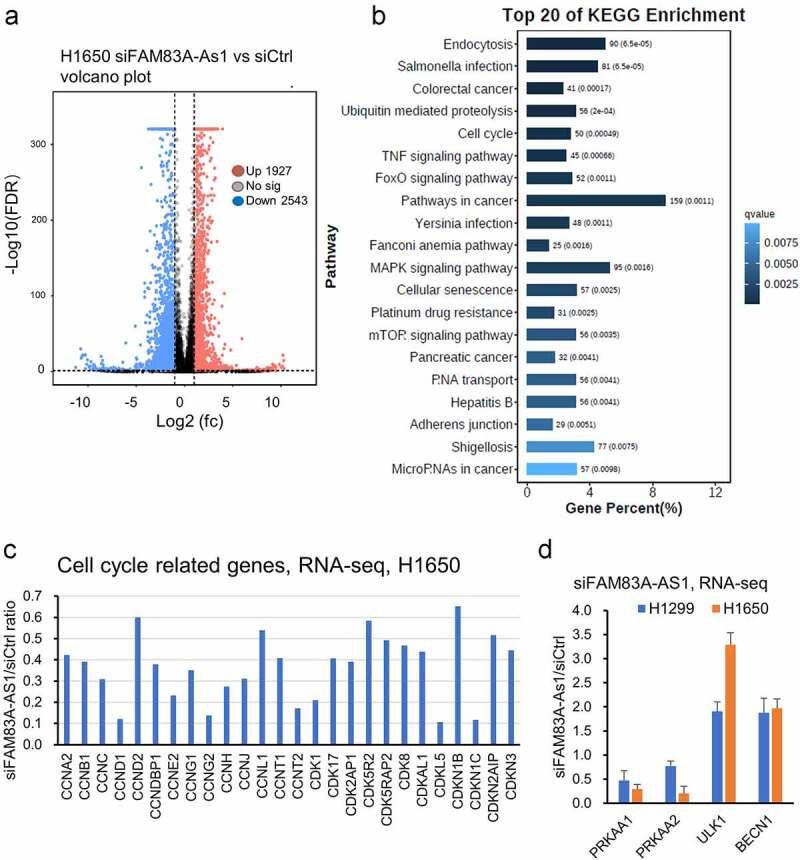
RNA-seq analysis showing *FAM83A-AS1* is involved in multiple cancer pathways. (a), Volcano plot showing the different expressed genes. As compared to siCtrl, there are 2543 down regulated and 1927 up regulated genes in siFAM83A-AS1 treated H1650 cell line. The cut off value are log2 of fold change (fc) ≤ −1 or ≥ +1, FDR 0.05 and PFKP value ≥ 0.3. (b), Top 20 KEGG pathway enrichment analysis using 2543 down regulated and 1927 up regulated genes in siFAM83A-As1 treated H1650 cell line. (c), Cell cycle related genes were down regulated after *FAM83A-AS1* knockdown with siRNAs in H1650 cells. (d), Changes of autophagy related genes upon *FAM83A-AS1* knockdown in H1650 and H1299 cells.

To uncover more biologic roles of *FAM83A-AS1* in lung cancer, we performed RNA-seq analysis after knockdown of *FAM83A-AS1* with siRNAs in H1650 and H1299 lung cancer cell lines. Volcano plots with the parameter of false discovery rate (FDR) below 0.05 and absolute fold change ≥2 were considered as differentially expressed genes. There are 2543 down regulated and 1927 upregulated genes in siFAM83A-AS1 treated H1650 cells (Figure 5(a)). We performed KEGG pathway analysis using these 4470 genes and found cell cycle pathway and autophagy-related mTOR signaling were on the top list, which further confirmed that *FAM83A-AS1* was involved in the cell cycle and autophagy regulation (Figure 5(b)). Figure 5(c) shows the cell cycle-related 26 genes (cyclins and cyclin-dependent kinases) decreased upon *FAM83A-AS1* knockdown in H1650 cells. Interestingly, *PRKAA1* and *PRKAA2* mRNAs (AMPKa protein-coding genes) were decreased upon *FAM83A-AS1* knockdown in both H1650 and H1299 cells (Figure 5(d)) although AMPKa protein was increased upon *FAM83A-AS1* knockdown (Figure 4(e,f)). *ULK1* and *BECN1* mRNAs were increased after *FAM83A-AS1* knockdown (Figure 5(d)). We have also found that 159 genes were involved in pathways in cancer (Figure 5(b)). Other pathways such as ubiquitin-mediated proteolysis, TNF signaling, FoxO signaling, MAPK signaling, and microRNAs in cancer were also on the top 20 KEGG pathways (Figure 5(b)).

The GO analysis was performed using these 4470 genes. From DO (Disease Ontology) enrichment analysis, we found that there are 1067 genes involved in cancer progression (Fig. S1). Biological process enrichment analysis showed that the cell cycle was on the top list (Fig. S2). Molecular function analysis indicated that protein binding was on the top list (Fig. S3). All these results suggest that *FAM83A-AS1* plays a critical role in cancer progression underlying multiple signaling regulations. More detailed mechanisms will need to be further discovered.

### 3.6. MET, EGFR, and AMPKα were regulated by FAM83A-AS1

**Figure 6.**
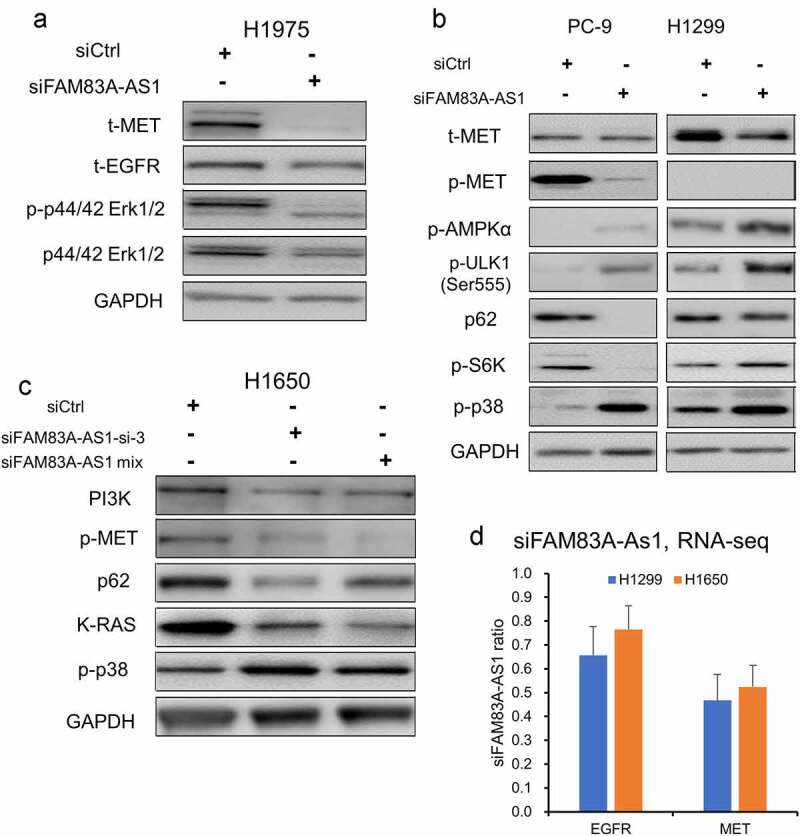
MET, EGFR and AMPKα were decreased upon *FAM83A-AS1* knockdown. (a–c), Silencing of *FAM83A-AS1* by siRNAs, MET, EGFR, ERK, PI3K and K-RAS proteins were down regulated in H1975, H1650, H1299 and PC9 cells. The autophagy related proteins p62 and phosphor S6K were also decreased in PC-9 and H1650 cells. (d), *MET* and *EGFR* mRNAs were decreased upon *FAM83A-AS1* knockdown in H1650 and H1299 cells measured by RNA-seq.

Since *FAM83A-AS1* play roles in lung cancer cell proliferation, migration and autophagy, we want to know the underlying molecular mechanism. RNA-seq analysis indicated *FAM83A-AS1* was involved in multiple cancer pathways (Figure 5). We firstly screened the EGFR and MET signaling, which are the most important pathways in lung cancer progression by Western blot. We found that, after knockdown *FAM83A-AS1*, serval oncogenic proteins including MET, EGFR, ERK1/2, PI3K, and K-RAS were decreased, while p38 increased in serval lung cancer cell lines (Figure 6(a–c)). RNA-seq analysis after knockdown of *FAM83A-AS1* showing that the mRNA levels of MET and EGFR were decreased more than 30–50% as compared to siCtrl in H1299 and H1650 cells (Figure 6(d)). These results suggest that *FAM83A-AS1* promotes cancer progression may through MET/EGFR signaling at the transcription levels. The detailed interaction among them will need further investigation.

Meanwhile, autophagy promoter proteins p-AMPKα and p-ULK1 were increased upon *FAM83A-AS1* knockdown in PC-9 and H1299 (Figure 6(b)). While, autophagy-related marker p62 and the autophagy inhibitor phosphor-S6K proteins were decreased in PC-9 and H1650 cells (Figure 6(b,c)) upon *FAM83A-AS1* knockdown, all these results further confirmed that *FAM83A-AS1* is involved in the regulation of autophagy.

**Figure 7.**
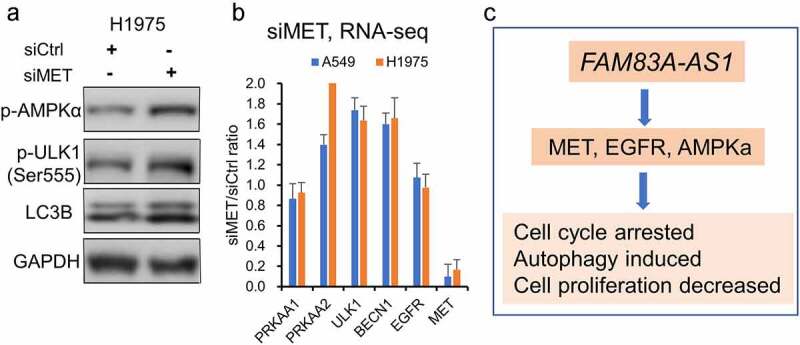
P-AMPKα was increased upon MET knockdown. (a), p-AMPK, p-ULK and LC3B II protein were increased upon *MET* knockdown in H1975 cells. (b), *PRKAA2, ULK1* and *BECN1* mRNAs were increased upon *MET* knockdown in H1975 and A549 cells measured by RNA-seq. *PRKAA1* and *EGFR* mRNAs were not changed. (c), the schematic of *FAM83A-AS1*-MET-AMPK signaling in regulating tumor progression and autophagy in lung adenocarcinoma.

We have found that AMPKa, EGFR, and MET were regulated by *FAM83A-AS1*. Since AMPKa is a well-known autophagy regulator [34] and MET is reported to play an important role in cancer progression [35], we want to know if MET can regulate AMPKa. To explore whether MET is involved in the underlying mechanism of *FAM83A-AS1* in regulating autophagy, Western blot analysis was performed following siRNA-mediated knockdown of *MET*. We found that p-AMPKα, p-ULK1, and LC3B II proteins were increased upon *MET* silencing in the H1975 cell line (Figure 7(a)). We also performed RNA-seq analysis upon *MET* gene knockdown by siRNAs. We found that *PRKAA2* (AMPKa coding gene 2), *ULK1*, and *BECN1* mRNAs were increased upon MET knockdown in H1975 and A549 cells (Figure 7(b)). *PRKAA1* (AMPKa coding gene 1) and *EGFR* mRNAs were not changed upon MET knockdown. Taking together, *FAM83A-AS1* regulates cancer progression and autophagy may via MET-AMPKα signaling in lung cancer (Figure 7(c)).

## 4. Discussion

LncRNAs were reported to have critical roles in cancer immunity, cancer metabolism, cancer metastasis, and drug resistance [36–39]. *FAM83A-AS1* has been found to play an oncogenic role in several cancers including esophageal, liver, and lung cancer [18–25]; however, the underlying mechanism of this lncRNA is not clear. In this study, we found that lncRNA *FAM83A-AS1* expression was up-regulated in lung cancer, and increased expression of this lncRNA was significantly related to poor patient survival. Functional investigation indicated that knockdown of *FAM83A-AS1* can decrease cell proliferation, migration, and invasion. The recently reported study is consistent with the results of our experiments and confirms that *FAM83A-AS1* is associated with EMT in lung cancer cells [40]. We further found that knockdown *FAM83A-AS1* induced autophagy in lung cancer cells. Mechanistically, *FAM83A-AS1* promotes tumor progression and autophagy may through MET-AMPKɑ signaling in lung adenocarcinoma.

So far, there are only several reports examining the expression and roles of lncRNA *FAM83A-AS1* in cancers. Huang *et al*. reported that *FAM83A-AS1* levels were higher in 62 squamous esophageal cancers and higher expression was correlated with poorer patient survival [18]. He *et al*. reported that *FAM83A-AS1* expressions were increased in 60 hepatocellular carcinomas [19]. Through the analysis of TCGA RNA-Seq data, Xiao and Shi groups [20,21] found that the level of *FAM83A-AS1* was upregulated in both LUAD and LUSC and higher expression was related to unfavorable survival in LUAD. Here, we analyzed the *FAM83A-AS1* expression not only in the TCGA RNA-Seq data used in Xiao and Shi’s studies [20,21] but also in another two RNA-Seq data sets including UM (67 LUAD) [12,29] and Seo (85 LUAD) [28] datasets, as well as an independent validation data set (101 LUAD) with the qRT-PCR assay. We found that the expression of *FAM83A-AS1* was higher in all four data sets with AUC 0.93–0.97, and higher expression was related to poor patient survival in our qRT-PCR validation set. In addition, we found *FAM83A-AS1* levels were higher in LUSC and LLC. Further, through the analysis of the CCLE lung cancer cell line’s RNA-Seq data [30], we confirmed that the *FAM83A-AS1* level was higher in LUAD, LUSC, and LLC, but not higher in SCLC. This suggests a cell-type specific role although the basis for this remains unknown. We found *FAM83A-AS1* expression was mainly located in cytoplasm which may represent some of where it influences cell function and a finding consistent with Xiao’s report [20].

*FAM83A-AS1* plays oncogenic roles in esophageal, liver, and lung cancers [18–21,23–25]. We found that knockdown of *FAM83A-AS1* decreased the cell proliferation, colony formation, invasion, and migration in H1299 and H838 lung cancer cells, which were consistent with others [18–21,40]. In addition, we found that the cell autophagy was induced upon *FAM83A-AS1* knockdown in H838 cells, which may be related to AMPK-MET signaling. The relationship between *FAM83A-AS1* and autophagy has never been reported.

The molecular mechanisms of *FAM83A-AS1* in promoting lung cancer progression is not clear. We found that serval oncogenic proteins including MET, EGFR, ERK1/2, PI3K, and K-RAS were down-regulated upon *FAM83A-AS1* knockdown. RNA-seq analysis after knockdown of *FAM83A-AS1* also indicated *FAM83A-AS1* involved in multiple cancer pathways. MET, also known as c-MET or the Receptor of Hepatocyte Growth Factor (HFG), is a tyrosine kinase and proto-oncogene located on chromosome 7q31.2 [41,42]. Its dysregulation in lung cancer was first discovered in the 1990s [43,44]. Since then, further research has examined MET signaling pathways in cancers [45–47]. Changes in MET have become a classic signal pathway for lung cancer progression including cell proliferation, migration, invasion, and tumor growth in our previous studies and others [11,17,35]. Here, we uncover that both MET protein and mRNA were decreased upon silencing of *FAM83A-AS1*, which suggests a possible connection between the oncogenic role of *FAM83A-AS1* and MET signaling. Furthermore, we found that AMPKɑ expression was regulated by MET.

## 5. Conclusions

Our study revealed that *FAM83A-AS1* expression was higher in lung cancer and associated with unfavorable patient survival, which has potential as a diagnosis/prognosis marker. *FAM83A-AS1* silencing can impair cell proliferation, invasion, migration, and colony formation, as well as induce cell autophagy. These cancer progression and autophagy regulation of *FAM83A-AS1* may be via MET-AMPKɑ signaling, which can lead to potential targeting for lung cancer therapy.

## Supplementary Material

Supplemental Material

## Data Availability

The datasets supporting the conclusions of this article are included within the article.
