# Supplementary material for: FAM83A antisense RNA 1 (FAM83A-AS1) silencing impairs cell proliferation and induces autophagy via MET-AMPKɑ signaling in lung adenocarcinoma
Source: Bioengineered. 2022 May 29;13(5):13312–27. doi: 10.1080/21655979.2022.2081457 (PMC9275865; doi:10.1080/21655979.2022.2081457)

Supplementary data

FAM83A Antisense RNA 1 (*FAM83A-AS1*) silencing impairs cell proliferation and induces autophagy via MET-AMPKα signaling in lung adenocarcinoma

Huijie Zhao, …, Guoan Chen

Figure S1

Disease Ontology Enrichment Analysis

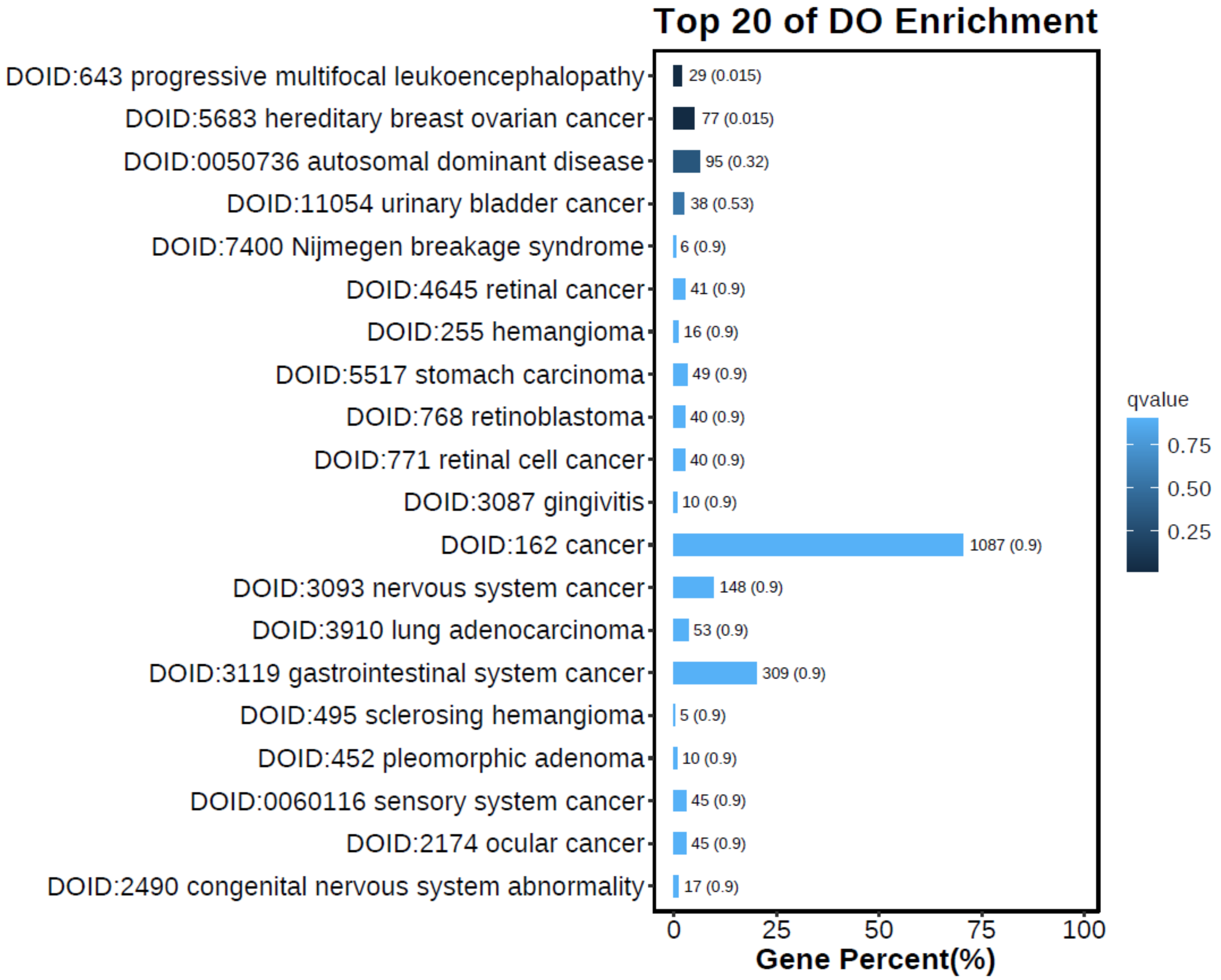

Figure S2

Biological Process Enrichment Analysis

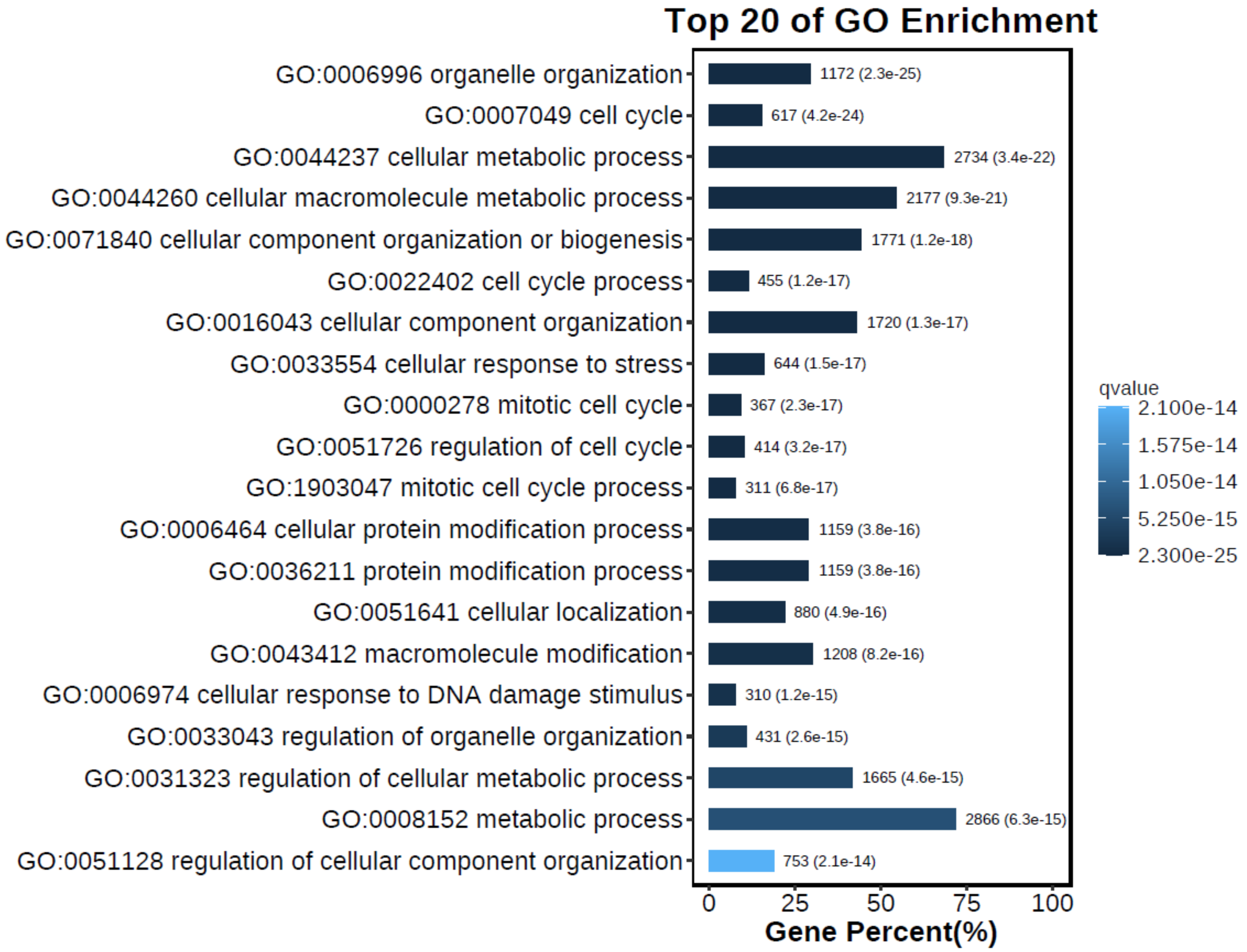

Figure S3

Molecular Function Enrichment Analysis

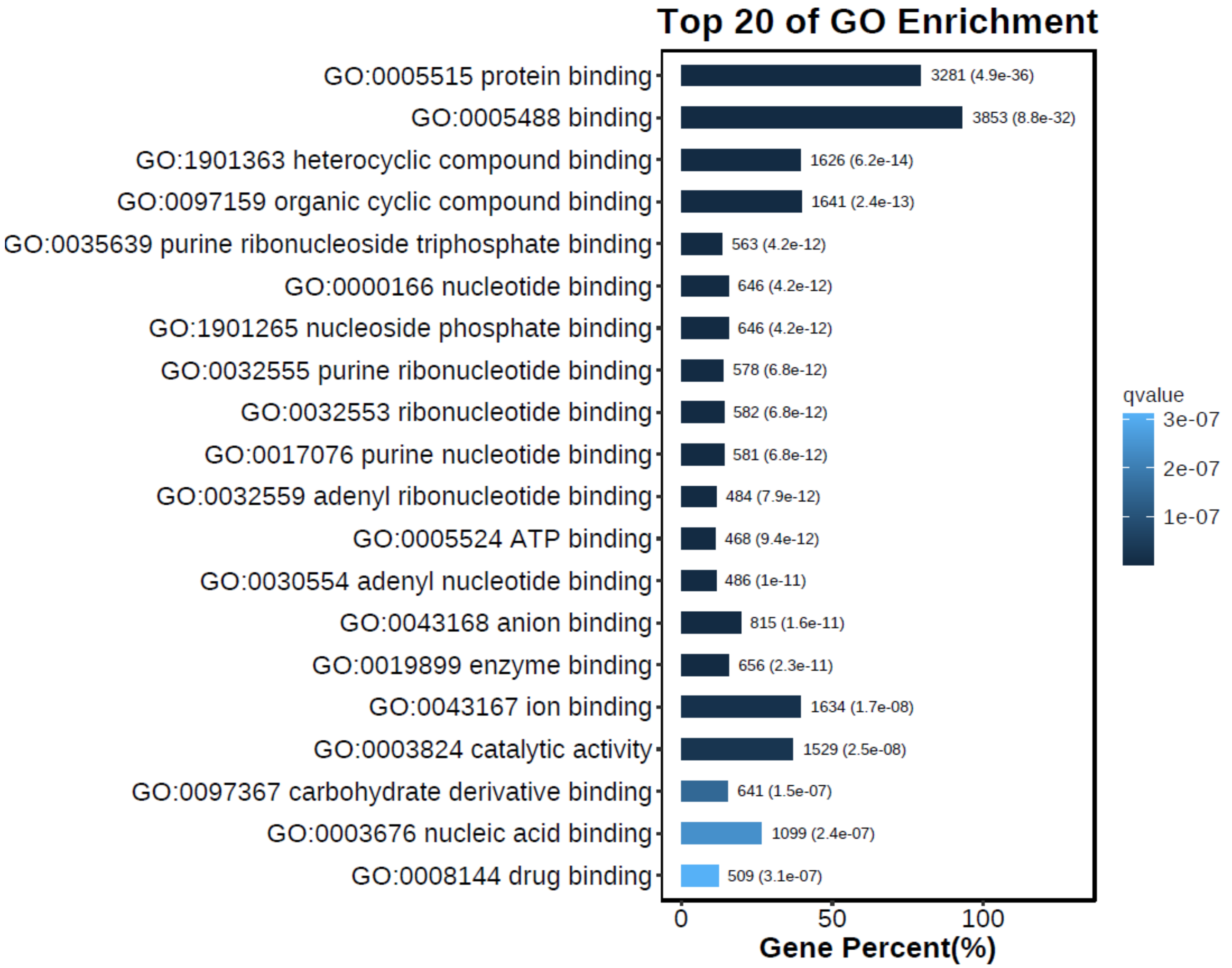

Supplement: Supplemental Material [file KBIE_A_2081457_SM5822.pdf]
